# Assessing awareness and attitude of Egyptian medical students towards emergency medicine as a specialty and career choice: A single-institutional study

**DOI:** 10.1016/j.afjem.2022.12.003

**Published:** 2023-01-12

**Authors:** Mohamed A Hussein, Sherif E AbdelMawgoud, Mostafa H Abd El Wahab, Mostafa Nagy, Mohamed El-Shinawi

**Affiliations:** a Faculty of Medicine, Ain Shams University, 38 Abbassia Square, Next to Al-Nour Mosque Cairo, Egypt 11517; b Department of General Surgery, Faculty of Medicine, Ain Shams University, 38 Abbassia Square, Next to Al-Nour Mosque Cairo, Egypt 11517

**Keywords:** Medical student, Awareness, Attitude, Emergency medicine, Specialty, Career choice

## Abstract

**Introduction:**

Emergency medicine (EM) was formally recognized as a specialty in Egypt in 2002. Many institutions of higher education do not yet have an operational academic department of emergency medicine. This study attempts to quantify the awareness and attitude of Ain Shams University medical students towards emergency medicine as both a specialty and a career.

**Methods:**

A paper-based survey was delivered to undergraduate medical students at the Faculty of Medicine, Ain Shams University in Cairo, Egypt between December 2021 and April 2022. The survey was designed to assess awareness towards the scope of practice of emergency physicians as well as general attitude toward emergency medicine as a specialty and career choice.

**Results:**

A total of 391 students and interns/house officers participated in this study. 53.2% of participants were females and the mean age was 21.65 ± 2.25 years. Only 30 participants (7.7%) were classified as having “Excellent knowledge” of emergency medicine, 92 (23.5%) as “Good knowledge”, 158 “40.4%” as “Fair knowledge” and 111 (28.4%) as “Poor knowledge”. The difference in scores between academic years was not statistically significant (p = 0.239). 91.8% of respondents favored the creation of student interest groups in EM and 40% of respondents found it difficult to reach information regarding EM.

**Conclusion:**

Our study demonstrates a lack of awareness and knowledge towards emergency medicine as a specialty across all academic years at our institution. Formal recognition of EM as a specialty doesn't guarantee widespread knowledge among medical students, particularly at institutions without academic EM departments.

## African relevance

• As more countries recognize emergency medicine as a specialty, it becomes necessary to shed light on areas that can impact the development effort.
• Recognition of emergency medicine as a specialty does not guarantee that a correct understanding of the specialty is widespread.
• Perceptions of students towards emergency medicine as a specialty must be continually assessed and awareness raised.

## Introduction

Emergency medicine (EM) is a relatively new specialty within the house of medicine [1]. In Egypt, emergency medicine was formally registered as a specialty in 2002. Many great strides have been made in the development of emergency medicine as a specialty in Egypt. There are multiple societies that aim to expand training in emergency and trauma care and subsequently enhance the quality of care provided in emergency centres in Egypt. Currently, medical graduates can pursue specialist certification in EM through two routes, either university-based training programs or programs facilitated by the Egyptian Ministry of Health and Population [2].

The Faculty of Medicine at Ain Shams University was founded in 1947, making it one of the oldest public faculties of medicine in Egypt. Although currently under development, the Faculty of Medicine at Ain Shams University, at the time of writing, does not yet have an operational academic department of emergency medicine, nor does it currently offer an undergraduate emergency medicine clerkship [3].

Many articles in the literature attempt to quantify the awareness, attitude, and perceptions of medical students towards emergency medicine as either a specialty or a career choice [4], [5], [6], [7], [8], [9], [10]. For example, Ray et al states that early exposure to EM positively impacted medical students’ choice of EM as a career [8]. This was also reported by Gharahbaghian et al whom found that exposure to the EM department strongly impacted students’ interest and decision to pursue EM as a career [10]. Furthermore, Cevik et al found that a mandatory EM clerkship enhanced students’ perceptions towards EM as a career [9]. Moreover, Adeyeye et al found poor awareness and overall knowledge about EM in Nigerian medical students [4]. To our knowledge, there have been no attempts to make this quantification in Egypt. Since medical students are naturally on the path to become physicians, it is important to assess their knowledge and general awareness towards future specialty choices.

Due to plans to both establish an academic department of emergency medicine and include an undergraduate emergency medicine and trauma clerkship in the final year at the Faculty of Medicine at Ain Shams University, we thought it was prudent to establish a baseline of both awareness and attitude towards emergency medicine as a specialty and as a career choice [11]. We hypothesized that medical students at the Faculty of Medicine at Ain Shams University would have poor knowledge of emergency medicine as a specialty largely due to the novelty of the specialty in Egypt and the absence of educational infrastructure pertaining to the specialty at the Faculty of Medicine at Ain Shams University.

## Methods

Between December 2021 and April 2022, we performed a cross-sectional observational study involving medical students enrolled at the Faculty of Medicine at Ain Shams University. Prior to the current format that was adopted in 2017, the undergraduate MBBCh program ran for a total of six years with one mandatory house officer training year. The first three years encompassed the pre-clinical basic science portion and the latter three years, the clinical portion of the program with clerkships in multiple medical and surgical specialties. The undergraduate MBBCh program is currently five years long with two mandatory house officer training years, and the revamped curriculum has adopted an entirely integrated and modular design that relies on the *integration* of material from the different disciplines of basic medical science into one module that addresses a particular system of the human body.

Before beginning the study, we developed a 23-item survey that consisted of 18 Likert-scale questions assessing general attitude towards emergency medicine as a specialty, clinical knowledge, clinical skills as well as information availability and attitude to emergency medicine as a career. The survey also contained 5 yes-no questions assessing availability of avenues to learn about emergency medicine. We also asked participants their age, sex, and academic year.

We validated the survey using face-validation from a group of experts that included a combination of international and Egyptian faculty members as well as experts on question design. We then piloted the survey on two stages during the period from March 2021 to October 2021. In the first stage, students from different school years were invited through social media to fill the survey which was delivered by SurveyMonkey due to COVID restrictions. Respondents were interviewed by phone to further ascertain question comprehension. The survey was modified to reflect feedback and then the second stage was carried out by sharing a Google Forms link on social media. A total of 65 responses were gathered in the second stage. The Cronbach's alpha for the pilot questionnaire was 0.704.

Approval was obtained from the research ethics committee at the Faculty of Medicine at Ain Shams University. We recruited a data collection team that was trained on how to collect data using this survey as well as the rationale behind each question. We also provided each surveyor with a standardized document containing a translation of each question in the Egyptian Arabic dialect in case the English version of the question was difficult to comprehend.

We opted for a face-to-face delivery method through printed surveys that were distributed to medical students randomly present on campus on any given day. Verbal informed consent was obtained before collecting the response. A full list of institutional e-mails of the students and interns/house officers wasn't accessible, so an online delivery method risked disseminating the questionnaire to an undefined study population, as well as problems relating to respondent's bias.

We studied medical students of all academic years enrolled in the MBBCh program at the Faculty of Medicine at Ain Shams University as well as house officers undertaking their national mandatory house officer training year. We calculated the sample size (n= 374) using Raosoft Sample Size Calculator [12] based on the generous assumption that each of the 7 classes contained a maximum of 2000 medical students with a total population size of 14,000 medical students. Additionally, we expected a 5% loss of response rate thus the final sample was calculated at 393 respondents.

Statistical analysis was performed using SPSS (Version 26.0). Categorical variables were analysed using Chi-square test, Fischer's exact test, or the Kruskal Wallis test when needed. We also used Student's t-test and one-way ANOVA for comparison of means. A p-value <0.05 was considered as statistically significant.

Two approaches were used in this analysis, questions covering knowledge about EM (Q1-Q13) were handled separately from those reporting baseline characteristics and attitude towards EM. Total scores for participants were derived from these 13 questions, which necessitated considering them as a single and continuous entity. The remaining questions were considered as standalone items as they assessed attitude and access to information about the specialty within the Egyptian context.

For the first 13 questions, each respondent score was calculated with a maximum score of 65. We considered four 4 arbitrary cut points to further analyse the findings. A respondent score above 50 was considered as having “Excellent knowledge”, 46-50 as having “Good knowledge”, 40-45 as having “Fair knowledge”, and finally less than 40 as having “Poor knowledge”.

For 391 participants, 5075 cells were entered successfully from a total of 5083 required for Q1 to Q13, with 14 cells (0.003%) being missing. Missing data were distributed in 9 questions (Q1, Q4, Q6, Q7, Q8, Q9, Q10, Q12 and Q13). Little's MCAR test was performed (p=0.003), showing data not missing completely at random. We considered multiple imputations for these missing items. Several plausible datasets were created, and results were combined from each of them. The remaining items, including Q14-Q23 as well as “Age”, “Sex”, and “Academic Year”, had a total of 20 missing cells (0.004%) out of 5083 required cells, with “Age” being the most missed item (7 cells). No imputations were considered for these items.

## Results

A total of 391 respondents contributed to this study. Female participants represented 53.2% of the study population. Mean age was 21.65 ± 2.25 years, ranging between 18 and 27 years. We included students across the six years of medical training in the Faculty of Medicine at Ain Shams University as well as freshly graduated, medical interns/house officers. First year students represented the largest stratum with 62 participants (15.9%), second year students were 59 (15.1%), third year students were 61 (15.6%). Fourth year students were our smallest stratum with 42 participants (10.7%), fifth year students were 59 participants (15.1%), while sixth year students were our second smallest stratum with 47 respondents (12%). 61 interns/house officers participated in this study representing 15.6% of the total study cohort. One hundred nine respondents (27.9%) replied that they were able to find official student activities for exploring EM. Three hundred fifty nine respondents (91.8%) answered that they would like faculty administration to facilitate the creation of student interest groups in EM.

**Fig. 1:**
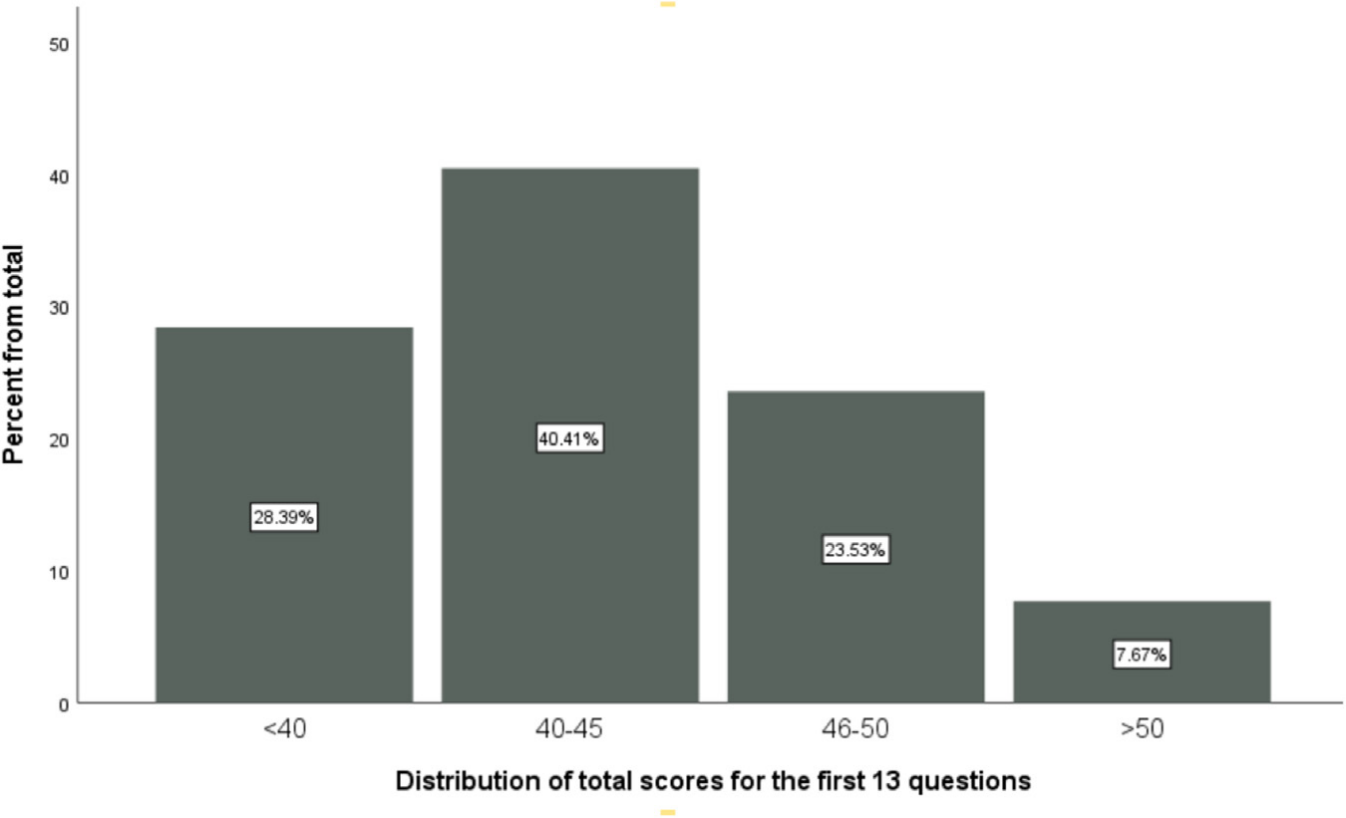
Distribution of total scores among different knowledge categories.

The first 13 questions are designed to reflect knowledge about EM, with a total scoring of 65 for the combined 13 questions. Details of the scoring process are provided in Table B.1. The mean score for the 13 questions was 43.38 ± 5.03 for the total study cohort, with males and females having comparable mean scores (43.27 ± 5.15 and 43.52 ± 4.33, respectively; p = 0.605). 111 participants (28.4%) were subsequently categorized as having “Poor knowledge” of EM, 158 (40.4%) as having “Fair knowledge”, 92 (23.5%) as having “Good knowledge” and only 30 (7.7%) as having “Excellent knowledge”. It is to be noted that the “Excellent knowledge” category was kept loose (i.e., participants having scores anywhere between 50 and 65 were considered to have excellent knowledge) due to the limited numbers in this stratum. Fig. (1) gives a graphical representation of these findings. Table 1 summarizes the differences between different academic years regarding mean scores and different scoring categories described previously, neither of which was statistically significant (ANOVA p-value = 0.239 and Kruskal Wallis test p= 0.321).

**Table 1:** Mean scores among different academic years and score categories distribution.

Academic Year	Mean score (±SD)	ANOVA for means	Score categories number (percent)				Total	Kruskal-Wallis
			Poor <40	Fair 40-45	Good 46-50	Excellent >50		

**1**	42.69 ± 5.1	F= 1.339 **p = 0.239**	22 (35.5%)	24 (38.7%)	10 (16.1%)	6 (9.7%)	62 (100%)	**p = 0.321**
**2**	42.05 ± 4.18		17(28.8%)	28 (47.5%)	10 (16.9%)	4 (6.8%)	59 (100%)	
**3**	42.6 ± 5.14		23(37.7%)	18 (29.5%)	16 (26.2%)	4 (6.6%)	61 (100%)	
**4**	43.61 ± 4.07		12 (28.6%)	15 (35.7%)	13 (31%)	2 (4.8%)	42 (100%)	
**5**	44.07 ± 4.5		12 (20.3%)	23 (39%)	21 (35.6%)	3 (5.1%)	59 (100%)	
**6**	43.17 ± 4.78		14 (29.8%)	21 (44.7%)	8 (17%)	4 (8.5%)	47 (100%)	
**Interns/** **House Officers**	44.5 ± 5.03		11 (18%)	29 (47.5%)	14 (23%)	7 (11.5%)	61 (100%)	
**Total**	43.38 ± 4.75	-	111 (28.4%)	158 (40.4%)	92 (23.5%)	30 (7.7%)	391 (100%)	-

These 13 questions are further considered to cover 3 different domains. The first 5 questions (Q1-Q5) cover general knowledge regarding emergency medicine as a specialty, (Q6-Q9) cover required clinical knowledge, and the last four questions (Q10-13) cover required clinical skills and capabilities of EM physicians.

To gain deeper insight into the data, we classified different academic years into two categories; the first one involved pre-clinical students (first to third year) and the second involved clinical students (fourth to sixth years in addition to interns/house officers). The rationale is that the first group's curriculums are concerned mainly with basic sciences while the second group's curriculums are more clinically oriented. We tested both groups (pre-clinical and clinical) regarding the 3 different domains listed in Table 2.

**Table 2:** Pre-clinical and clinical differences regarding different domains.

Domain	Category (Number)	Mean (±SD)	Independent t-test
**General knowledge regarding EM as a specialty**	**Pre-clinical** (182)	15.33 ± 2.25	p = 0.205
	**Clinical** (209)	15.63 ± 2.4	
**Clinical Knowledge**	**Pre-clinical** (182)	13.48 ± 2.45	**p =0.003**
	**Clinical** (209)	14.19 ± 2.27	
**Clinical Skills**	**Pre-clinical** (182)	13.97 ± 2.85	p = 0.67
	**Clinical** (209)	14.08 ± 2.36	

Questions 14 to 18 have no true scoring. They cover the general attitude of the participants towards EM as a career within the Egyptian context. To maintain statistical power, “Agree” and “Strongly agree” were combined into “Generally agree”. “Disagree” and “Strongly disagree” were also combined into “Generally disagree”. Chi-Squared tests were used to examine the weight of these three proportions among different academic years. Detailed responses in every academic year are provided in Table C.1. A summary of results is provided in Table 3.

**Table 3:** General attitude and information accessibility.

Question	Response	Number (percentage)	Chi-squared among academic years
**Shift work provides enough financial compensation for emergency physicians in Egypt relative to other specialties.**	**Agree**	102 (26.2%)	**p = 0.002**
	**Neutral**	137 (35.1%)	
	**Disagree**	151 (38.7%)	

**Inability to operate a private clinic is a disadvantage for emergency physicians.**	**Agree**	220 (56.4%)	**p = 0.001**
	**Neutral**	112 (28.7%)	
	**Disagree**	58 (14.9%)	

**You will pursue residency training in emergency medicine.**	**Agree**	139 (35.8%)	p = 0.05
	**Neutral**	145 (37.4%)	
	**Disagree**	104 (26.8%)	

**You need more information to make a decision about pursuing residency training in emergency medicine.**	**Agree**	271 (69.3%)	p = 0.119
	**Neutral**	84 (21.5%)	
	**Disagree**	36 (9.2%)	

**It is easy to access information about practicing emergency medicine in Egypt.**	**Agree**	76 (19.4%)	**p = 0.001**
	**Neutral**	158 (40.4%)	
	**Disagree**	157 (40.2%)	

In the first question interns/ house officers were the most suspicious regarding abilities of shift work to provide financial compensation, scoring the highest Disagree/ Agree ratio (24:14). In the second question, interns/ house officers, along with third year students, saw that inability to operate a private clinic was a major drawback, both scoring the highest Agree/ Disagree ratio (42:5). In the third question, first year students had the highest number of aspiring emergency physicians, scoring the highest Agree/ Disagree ratio (27:11) compared to medical interns/ house officers who had the lowest Agree/ Disagree ratio (12:18). In the fourth question, respondents were more uniform in their answers aiming for more information to decide. In the fifth question, interns/ house officers found it difficult to access information about EM in Egypt, scoring the highest Disagree/ Agree ratio (33:7), compared to first year (24:17) and second year students (9:18).

Participants were asked if they personally know any practicing emergency physicians; 280 participantss (71.6%) responded with “No” compared to 111 (28.4%) who responded with “Yes”. Participants were also asked if they had looked for information about EM as a specialty during their studies; 228 (58.3%) answered with “No” compared to 163 (41.7%) who answered with “Yes”.

## Discussion

To our knowledge, this is the first study to assess the knowledge, attitude, and/or awareness towards EM in a single institution in Egypt. In addition, other than the study by Adeyeye et al. [4], there are no studies that measure these elements in low- and middle-income countries.

Of note, there are no formal student activities pertaining to EM education and awareness at the Faculty of Medicine at Ain Shams Univeristy. However, 27.9% of respondents reported that they were able to find official student activities that facilitated the exploration of EM. This may represent activities organized by informal bodies or general student activities promoting EM knowledge. Despite that, we found that most respondents (91.8%) would like faculty administration to facilitate the creation of an EM-related student activity. Only 76 (19.4%) participants agreed that it was easy to access information about practing EM in Egypt and 271 (69.3%) expressed that they needed further information to decide whether to pursue EM or not. This highlights the need to provide access to information about EM as well as exposure to the specialty.

Our study showed that there was no statictically significant difference in overall scores between students of different academic years regarding knowledge about EM. There is poor knowledge overall about EM across different academic years. Our findings are congruent with findings of Adeyeye et al on Nigerian medical students. The authors acknowledged poor overall knowledge of EM despite exposure to rotations and clinical activities (i.e., students in clinical years) probably due to lack of EM residency or post-graduate training and due to the supervision of emergency rooms by consultants of other specialties.

In our study, there was no statistically significant difference in scores between students in pre-clinical and clinical years regarding general knowledge of EM as a specialty or awareness of the required clinical skills for emergency physicians. However, clinical year students had higher scores (p=0.003) in the domain assesing their awareness of the required clinical knowledge for an emergency physician, probably due to more clinical exposure. Leung et al recruited medical students from year 3 to year 5 with variable exposure to the EM curriculum. It was found that fifth year students, with more exposure to EM, had a more positive attitude towards EM when compared to third and fourth year students [7].

Our participants were asked if they will pursue residency training in EM. Only 139 (35.8%) respondents mentioned that they will pursue residency training in EM (p=0.05). This may be explained by the strong association between high knowledge scores and willingness to pursue EM, described by Adeyeye et al., which supports the need for increasing awareness and knowledge of EM as a specialty amongst medical students. On the other hand, Leung et al showed that attitude score is not related to eventual choice of EM as a career specialty but rather the presence of other factors that intervene. In a study by Hillier et al of medical students in Canadian universities, authors mentioned that family life and control over work schedule were among the top influential factors regarding the decision to seek EM as a career choice [13]. In another study by Alkhaneen et al on Saudi medical students, hospital orientation (in-hospital care, urgent care, immediate outcome, prefer medical problems over social problems) and medical lifestyle (acceptable on-call schedule, research interest, less intense residency program) were the top influential factors to impact choice of EM while prestige was the lowest factor reported. High income and focus on urgent care are the top factors preferred by students choosing to pursue EM [5]. Chew et al reported that focus on urgent care, salary, and exposure to elective EM training influenced junior students to seek EM as a career choice. Senior students were more likely to pursue EM due to exposure to elective EM training [14].

Our study has several limitations. First, the study is conducted on medical students at our institution and the results do not necessarily apply to other medical students at other institutions in Egypt. Secondly, we did not assess the factors that might influence choice of EM as we hypothesized that most medical students lack sufficient knowledge regarding EM as both a specialty and career. As previously mentioned, medical students were studying according to multiple curricular designs. This may have influenced perceived clinical exposure as it occurs earlier in the new modular program. Furthermore, our sampling method can be classified as convenience sampling which may introduce sampling bias. Also, the subjective nature of the score cut-off points can be considered a limitation despite being purposely lax with what constituted excellent knowledge. Finally, our study is a cross sectional study with no prospective data on medical students’ eventual specialty choice.

In conclusion, our study suggests that formal recognition of EM as a specialty does not guarantee that awareness and knowledge is widespread among the medical community, particularly among medical students at institutions without academic departments. Thus, establishment of academic departments and providing undergraduate clerkships as well as maintaining accessible sources of information are paramount in increasing awareness and shaping attitudes towards EM as a specialty and career choice. This provides students with the tools to make an informed decision about specialization and raises awareness about the role emergency physicians play in a healthcare system among students that ultimately decide to pursue careers in other specialties.

## Dissemination of results

Results from this study was shared with staff members at the data collection site through an informal presentation.

## Authors’ contribution

Authors contributed as follow to the conception or design of the work; the acquisition, analysis, or interpretation of data for the work; and drafting the work or revising it critically for important intellectual content: MAH contributed 50%; SEA 20%; MHA 20%, MN, MS contributed 5% each. All authors approved the version to be published and agreed to be accountable for all aspects of the work.

## Declaration of Competing Interest

The authors declared no conflicts of interest.

## Appendix Supplementary materials

Image, application 1

Image, application 2
