# Supplementary material for: Assessing awareness and attitude of Egyptian medical students towards emergency medicine as a specialty and career choice: A single-institutional study
Source: Afr J Emerg Med. 2023 Jan 12;13(1):20–4. doi: 10.1016/j.afjem.2022.12.003 (PMC9860339; doi:10.1016/j.afjem.2022.12.003)
Supplement: Supplementary file 1 [file mmc1.docx]

**Table B.1** Scoring of the first 13 questions.

| **Question** | **SA** | **A** | **N** | **D** | **SD** |
| --- | --- | --- | --- | --- | --- |
| The presence of a Department of Emergency Medicine and trained emergency physicians is necessary for optimal patient outcome. | 5 | 4 | 3 | 2 | 1 |
| Practicing emergency medicine is stressful all the time. | 1 | 2 | 3 | 4 | 5 |
| Emergency physicians can discharge patients and provide them with follow up plans. | 5 | 4 | 3 | 2 | 1 |
| Emergency physicians are non-specialized general practitioners. | 1 | 2 | 3 | 4 | 5 |
| The emergency department should be supervised by other specialties. | 1 | 2 | 3 | 4 | 5 |
| Emergency physicians should refer all fractures to the orthopedics department. | 1 | 2 | 3 | 4 | 5 |
| Emergency physicians should directly refer all cases of bleeding in pregnant women to the obstetrics and gynecology department. | 1 | 2 | 3 | 4 | 5 |
| Emergency physicians should know how to manage common toxicological syndromes. | 5 | 4 | 3 | 2 | 1 |
| Emergency physicians should know how to manage electrolyte disturbances. | 5 | 4 | 3 | 2 | 1 |
| Emergency physicians can expertly manage a patient’s airway. | 5 | 4 | 3 | 2 | 1 |
| Emergency physicians can perform advanced interventional procedures (e.g., ultrasound guided temporary transvenous cardiac pacing). | 5 | 4 | 3 | 2 | 1 |
| Emergency physicians should be able to use ultrasound in the workup of patients. | 5 | 4 | 3 | 2 | 1 |
| Emergency physicians can perform cesarean sections in pregnant patients suffering from cardiac arrest (i.e., perimortem cesarean section) | 5 | 4 | 3 | 2 | 1 |

SA= Strong Agree, A = Agree, N = Neutral, D= Disagree, SD = Strongly disagree.
