# Supplementary material for: Assessing awareness and attitude of Egyptian medical students towards emergency medicine as a specialty and career choice: A single-institutional study
Source: Afr J Emerg Med. 2023 Jan 12;13(1):20–4. doi: 10.1016/j.afjem.2022.12.003 (PMC9860339; doi:10.1016/j.afjem.2022.12.003)
Supplement: Supplementary file 2 [file mmc2.docx]

Table C.1 Academic years responses for questions covering general attitude towards EM as a career within the Egyptian context (Q14:18).

| **Question** | **Academic year** | **GA** | **GD** | **N** | **T** |
| --- | --- | --- | --- | --- | --- |
| Shift work provides enough financial compensation for emergency physicians in Egypt relative to other specialties. | 1 | 20 | 17 | 24 | 61 |
|  | 2 | 15 | 13 | 31 | 59 |
|  | 3 | 16 | 24 | 21 | 61 |
|  | 4 | 5 | 19 | 18 | 42 |
|  | 5 | 17 | 33 | 9 | 59 |
|  | 6 | 15 | 17 | 15 | 47 |
|  | Intern/ House officer | 14 | 28 | 19 | 61 |
| Inability to operate a private clinic is a disadvantage for emergency physicians. | 1 | 27 | 18 | 16 | 61 |
|  | 2 | 21 | 11 | 27 | 59 |
|  | 3 | 42 | 5 | 14 | 61 |
|  | 4 | 29 | 4 | 9 | 42 |
|  | 5 | 34 | 8 | 17 | 59 |
|  | 6 | 25 | 7 | 15 | 47 |
|  | Intern/ House officer | 42 | 5 | 14 | 61 |
| You will pursue residency training in emergency medicine. | 1 | 27 | 11 | 23 | 61 |
|  | 2 | 26 | 12 | 21 | 59 |
|  | 3 | 22 | 17 | 22 | 61 |
|  | 4 | 14 | 11 | 17 | 42 |
|  | 5 | 16 | 23 | 18 | 57 |
|  | 6 | 22 | 12 | 13 | 47 |
|  | Intern/ House officer | 12 | 18 | 31 | 61 |
| You need more information to make a decision about pursuing residency training in emergency medicine. | 1 | 38 | 7 | 17 | 62 |
|  | 2 | 45 | 1 | 13 | 59 |
|  | 3 | 39 | 5 | 17 | 61 |
|  | 4 | 36 | 3 | 3 | 42 |
|  | 5 | 39 | 10 | 10 | 59 |
|  | 6 | 32 | 4 | 11 | 37 |
|  | Intern/ House officer | 42 | 6 | 13 | 61 |
| It is easy to access information about practicing emergency medicine in Egypt. | 1 | 17 | 24 | 21 | 62 |
|  | 2 | 18 | 9 | 32 | 59 |
|  | 3 | 14 | 24 | 23 | 59 |
|  | 4 | 5 | 17 | 20 | 42 |
|  | 5 | 4 | 32 | 23 | 59 |
|  | 6 | 11 | 18 | 18 | 47 |
|  | Intern/ House officer | 7 | 33 | 21 | 61 |

GA = Generally agree, GD = Generally disagree, N = Neutral, T = Total.
